# Identification, gene expression and genetic polymorphism of zinc finger A20/AN1 stress-associated genes, *HvSAP*, in salt stressed barley from Kazakhstan

**DOI:** 10.1186/s12870-020-02332-4

**Published:** 2020-10-14

**Authors:** Akmaral Baidyussen, Maryam Aldammas, Akhylbek Kurishbayev, Malika Myrzabaeva, Askar Zhubatkanov, Grigory Sereda, Raisa Porkhun, Sergey Sereda, Satyvaldy Jatayev, Peter Langridge, Carly Schramm, Colin L. D. Jenkins, Kathleen L. Soole, Yuri Shavrukov

**Affiliations:** 1 Faculty of Agronomy, S. Seifullin Kazakh AgroTechnical University, Nur-Sultan, Kazakhstan; 2 College of Science and Engineering, Biological Sciences, Flinders University, Adelaide, SA Australia; 3 A.F. Khristenko Karaganda Agricultural Experimental Station, Karaganda, Kazakhstan; 4 Wheat Initiative, Julius-Kühn-Institute, Berlin, Germany

**Keywords:** Barley, Gene expression, Genetic polymorphism, *HvSAP*, Marker-assisted selection, SNP marker, Stress-associated proteins, Salinity, Zinc finger A20/AN1 transcription factor

## Abstract

**Background:**

A family of genes designated as the Zinc finger A20/AN1 Transcription factors encoding stress-associated proteins (*SAP*) are well described in *Arabidopsis* and rice, and include 14 *AtSAP* and 18 *OsSAP* genes that are associated with variable tolerances to multiple abiotic stresses. The *SAP* gene family displays a great diversity in its structure and across different plant species. The aim of this study was to identify all *HvSAP* genes in barley (*Hordeum vulgare* L.), to analyse the expression of selected genes in response to salinity in barley leaves and develop SNP marker for *HvSAP12* to evaluate the association between genotypes of barley plants and their grain yield in field trials.

**Results:**

In our study, 17 *HvSAP* genes were identified in barley, which were strongly homologous to rice genes. Five genes, *HvSAP5*, *HvSAP6*, *HvSAP11*, *HvSAP12* and *HvSAP15*, were found to be highly expressed in leaves of barley plants in response to salt stress in hydroponics compared to controls, using both semi-quantitative RT-PCR and qPCR analyses. The Amplifluor-like SNP marker KATU-B30 was developed and used for *HvSAP12* genotyping. A strong association (R^2^ = 0.85) was found between KATU-B30 and grain yield production per plant of 50 F_3_ breeding lines originating from the cross Granal × Baisheshek in field trials with drought and low to moderate salinity in Northern and Central Kazakhstan.

**Conclusions:**

A group of *HvSAP* genes, and *HvSAP12* in particular, play an important role in the tolerance of barley plants to salinity and drought, and is associated with higher grain yield in field trials. Marker-assisted selection with SNP marker KATU-B30 can be applied in barley breeding to improve grain yield production under conditions of abiotic stress.

## Background

A large group of stress-associated proteins (SAP) is encoded by zinc-finger proteins containing two conservative domains A20/AN1. In humans, these genes act as Tumor necrosis factor, TNF [1] but in plants, *SAP* genes play an important role in different types of abiotic and biotic stresses, and they are well known as a part of the plant immunity response (Reviewed in [2]). Widely present in plant species, *SAP* genes remain very conservative structurally but have very diverse functions [3]. Identified firstly in rice [4], 18 *OsSAP* genes were shown to have a strong similarity to 14 *AtSAP* genes of *Arabidopsis thaliana* [5]. *SAP* genes have also been studied in other plant species like maize [3], tomato [6], grasses *Festuca arundinacea* and *Leymus chinensis* [7, 8], wheat [9], as well as cotton [10], plum and apple [11, 12], *Brassica napus* and *Medicago truncatula* [13, 14]. However, barley (*Hordeum vulgare*), an important cereal crop, represents a gap in our knowledge in this area, with no *SAP* genes as yet described and studied.

Similar to humans, *SAP* genes in plants are involved in the pathogen-recognition process via membrane-bound receptor-like kinases [15], and in defence mechanisms against bacteria and fungi infections, as shown in the recent examples of tomato *SlSAP3* and *SlSAP4* silencing and over-expression [16, 17]. However, much stronger responses of *SAP* genes were found in plants in response to various abiotic stresses including drought, salinity, cold and others (Reviewed in [2]).

Regardless of plant species, different *SAP* genes have specific expression profiles in response to abiotic stresses. All 18 *OsSAP* genes in rice and 13 identified *SlSAP* genes in tomato were responsive to one or multiple stresses amongst the wide range of abiotic stresses applied [5, 6]. *AtSAP10* was reported as strongly involved in the tolerance of *Arabidopsis* plants to heavy metals and high temperature [18]. For salinity, most *SAP* genes in rice, *Arabidopsis* and tomato were responsive to NaCl at the seedling stage [5, 6], while 33 out of 37 *GhSAP* genes were expressed in one-month-old cotton plants after exposure for 2 h of strong salinity stress (300 mM NaCl) [10]. The same strong level of salinity applied to young plants of *Medicago truncatula* for 2–12 h resulted in up-regulation of four *MtSAP* genes and down-regulation of five other genes [14]. One-month-old plants of the perennial grass, *Leymus chinensis*, were exposed to an even higher concentration of NaCl (400 mM) for 6–24 h, and transcript levels of *LcSAP* continued to increase [8].

Drought is reported to trigger strong expression of *MdSAP15*, *MdSAP25* and *MdSAP29* genes in apple [12] but, in contrast, *OsSAP7* gene was down-regulated in rice shoots [19]. The expressions of *FaSAP* (closest homolog of *OsSAP8*) in the grass species, *Festuca arundinacea*, and *PpSAP1* in plum (*Prunus persica*) were also highly up-regulated in plants under either salinity or drought [7, 11]. Showing a comparable trend, two *SAP* genes (*BnaA03g47350D* and *BnaC07g39590D*), homologous to *AtSAP10* and *OsSAP3* / *OsSAP5*, were induced with salt and dehydration with PEG in roots of *Brassica napus* as determined by the RNA-Seq technique [13].

Over-expression of *AtSAP10*, *OsSAP7* and *MdSAP15* (closest homologs of *OsSAP4* / *OsSAP8*) transgenes in *Arabidopsis* were very beneficial in improving plant tolerance to heavy metals and drought, respectively [12, 18, 19]. Transgenic tobacco plants over-expressing *LmSAP* from ornamental alyssum, *Lobularia maritima*, showed high expression of the transgene, with A20 and AN1 domains specifically involved in salinity/osmotic and oxidative stress responses, respectively [20]. In transgenic wheat and rice with over-expressed *SAP*, either higher grain yield or no net effect under various abiotic stresses was reported (Reviewed in [2]).

Variability in *SAP* gene expression in response to abiotic stresses is well documented in various plant species (Reviewed in [2]) but very little information has been recorded where such expression is associated with haplotypes, mutations or genetic polymorphism. In this context, molecular markers can be designed to indicate strong or weak associations between a haplotype and expression of a particular *SAP* gene. For example, a strong association was reported between three molecular markers surrounding the *TaSAP1-A1* gene and agronomically important traits like 1000 grain weight, number of grains per spike, spike length, peduncle length and total number of spikelets per spike in 300 bread wheat accessions grown in both drought and well-watered conditions [9]. Among a wide range of molecular markers, SNP (Single nucleotide polymorphisms) are very popular, and the recently developed Amplifluor-like SNP markers [21] were selected and applied in the current study, chosen from a very diverse range of SNP detection methods (Reviewed in [22]).

In addition to natural variation, hybrid populations and their progenies can also be used for genetic analysis of a preferred haplotype of the *SAP* gene to indicate segregation pattern and the type of gene inheritance. Association analysis of the *SAP* genetic background and plant performance in a stressed environment can provide direct information supporting the role and function of the *SAP* gene.

Barley (*Hordeum vulgare* L.) is a diploid plant species from the grass family and Triticeae tribe. The barley haploid genome size is about 5.3 Gb, with seven chromosomes, making it one of the largest of the recently sequenced genome of diploid species to date [23]. Barley plants show some tolerance to several abiotic stresses and can grow in relatively harsh and unfavourable environments. However, the grain yield of barley could be improved with better understanding of the mechanisms for tolerance to such abiotic stresses as drought and salinity [24]. In the very complicated gene-regulatory network system of barley plants, *HvSAP* genes potentially play an important role in the coordinated response and signalling system combatting abiotic stresses.

In the current study, within the framework of an International collaborative project, as indicated in the Funding section, local barley cultivars from Kazakhstan and one generated hybrid were used. With such a large territory available for field crops in Kazakhstan, strong drought and high salinity are very typical of some areas. Barley seed is in strong demand for animal feed and, therefore, a better understanding of the genetic mechanisms of barley plants’ tolerance to abiotic stresses can aid not only local agri-business, but also be beneficial for researchers and breeders working with barley and other crops in many countries.

The aims of this study were: (1) to identify a comprehensive list of *HvSAP* genes present in the barley genome and to compare them with the reference genome of rice; (2) to study and select the *HvSAP* genes most responsive to salinity using semi-quantitative and qRT-PCR; (3) to search for natural polymorphisms of *HvSAP12* among parents of a barley hybrid population; (4) to develop an SNP marker for hybrid plant genotyping and marker-assisted selection; and (5) to evaluate the association between selected hybrid genotypes and their phenotypes for grain yield performance in the strong drought and low to moderate salinity-affected environments of Northern and Central Kazakhstan.

## Results

### Identification of *HvSAP* candidate genes using bioinformatics approaches

During screening of SNPs in the barley database (http://bioinf.scri.ac.uk/barley_snpdb), the SNP ID: ABC08579 was found as a potentially interesting candidate gene. The annotation of the closely related rice accession AAP37480, putative A20/AN1-type zinc finger protein in *Oryza sativa*, group *japonica* was indicated. A search for a barley homologous gene using IPK (https://webblast.ipk-gatersleben.de/barley_ibsc/viroblast.php) and NCBI databases (https://www.ncbi.nlm.nih.gov) revealed that the identified SNP is present in the *HvSAP12* gene encoding a Stress-associated protein. Similar to rice, *HvSAP12* belongs to a Transcription factor A20/AN1-type zinc finger protein group. The annotated barley genes HORVU2Hr1G053670 and AK363382 were identified, respectively, from the IPK and NCBI databases for *HvSAP12*, and 17 *HvSAP* genes were found in total in the barley genome, strongly homologous to rice genes (Table 1). Small rearrangements in the barley genome were evident, where two *HvSAP* genes were duplicated (*HvSAP9a* and *HvSAP17a*), but three other genes (*HvSAP8*, *HvSAP13* and *HvSAP18*) were lost compared to the 18 *OsSAP* genes in rice [5].

**Table 1:** List, annotation and distribution of *HvSAP* genes in the barley genome and their corresponding homologs in rice. The *HvSAP12* and homologs are indicated in Bold

Gene	Gene/protein ID in IPK database	Chromosome	Gene ID in NCBI database	Protein ID in NCBI database	Protein Uni Ref100	Gene ID in rice database
*HvSAP1*	HORVU5Hr1-G072920	5H	AK372328, AK357531	BAK03526, BAJ88745	A2Z2J5	LOC_Os09g31200
*HvSAP2*	HORVU3Hr1-G067990 MLOC_39637	3H	No match	No match	Q942F8	LOC_Os01g52030
*HvSAP3*	HORVU2Hr1-G042070	2H	No match	No match	Q5JN07	LOC_Os01g56040
*HvSAP4* (*HvSAP8*)	HORVU6Hr1-G033550	6H	AK354794, AK250311	BAJ86013	Q6H7P8, A2YEZ6	LOC_Os02g10200, LOC_Os06g41010
*HvSAP5*	MLOC_43986	7H	AK372340	BAK03538	Q6H754	LOC_Os02g32840
*HvSAP6*	HORVU5Hr1-G104240	5H	AK370302	BAK01503	Q852K5	LOC_Os03g57890
*HvSAP7*	HORVU5Hr1-G103940	5H	No match	No match	Q852K6	LOC_Os03g57900
*HvSAP9*	HORVU2Hr1-G022940 MLOC_73029	2H	No match	No match	Q7Y1W9	LOC_Os07g07350
*HvSAP9a*	HORVU2Hr1-G036250 MLOC_17636	2H	No match	No match	Q7Y1W9	LOC_Os07g07350
*HvSAP10* (*HvSAP9*)	HORVU5Hr1-G103980	5H	No match	No match	Q69LE0, Q7Y1W9	LOC_Os07g07400, LOC_Os07g07350
*HvSAP11*	HORVU7Hr1-G050270	7H	AK359310	BAJ90521	Q84PD8	LOC_Os08g39450
***HvSAP12*** (*HvSAP9*)	**HORVU2Hr1-G053670 MLOC_79442**	**2H**	**AK363382**	**BAJ94586**	**Q6Z541, Q7Y1W9**	**LOC_Os08g33880, LOC_Os07g07350**
*HvSAP14*	HORVU5Hr1-G104360	5H	No match	No match	Q852K8	LOC_Os03g57920
*HvSAP15*	HORVU1Hr1-G032850	1H	No match	No match	Q0DJC7	LOC_Os05g23470
*HvSAP16*	HORVU2Hr1-G038760	2H	AK367174, AK360983	BAJ98377	Q0D5B9	LOC_Os07g38240
*HvSAP17*	HORVU5Hr1-G059890	5H	AK370364	BAK01565	Q6H595	LOC_Os09g21710
*HvSAP17a*	HORVU5Hr1-G059900	5H	AK370364	BAK01565	Q6H595	LOC_Os09g21710

**Fig. 1:**
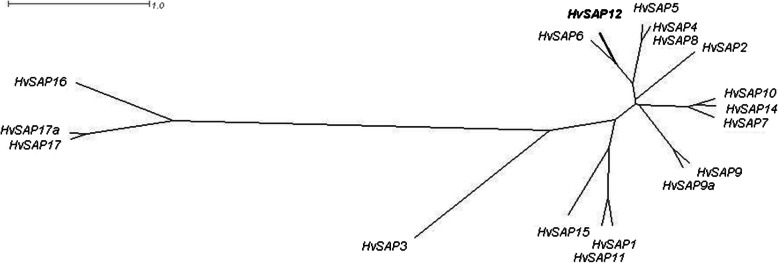
Molecular phylogenetic tree of 17 identified *HvSAP* genes based on their sequences in the barley genome. Unrooted Consensus tree with Equal angle dendrogram was generated by the program SplitsTree4 (http://www.splitstree.org). The position of the *HvSAP12* gene with the described SNP is indicated in Bold

A molecular phylogenetic tree of the 17 *HvSAP* genes (Fig. 1) was identical in order and pattern between the gene nucleotide and the deduced protein amino acid sequences. The analysis revealed the closest molecular similarity between *HvSAP12* and the *HvSAP6*, with relatively close similarity to *HvSAP5* and *HvSAP4/HvSAP8*. *HvSAP3* was isolated, but still closer than the most isolated group of *HvSAP16* and paired *HvSAP17* and *HvSAP17a* genes (Fig. 1).

**Fig. 2:**
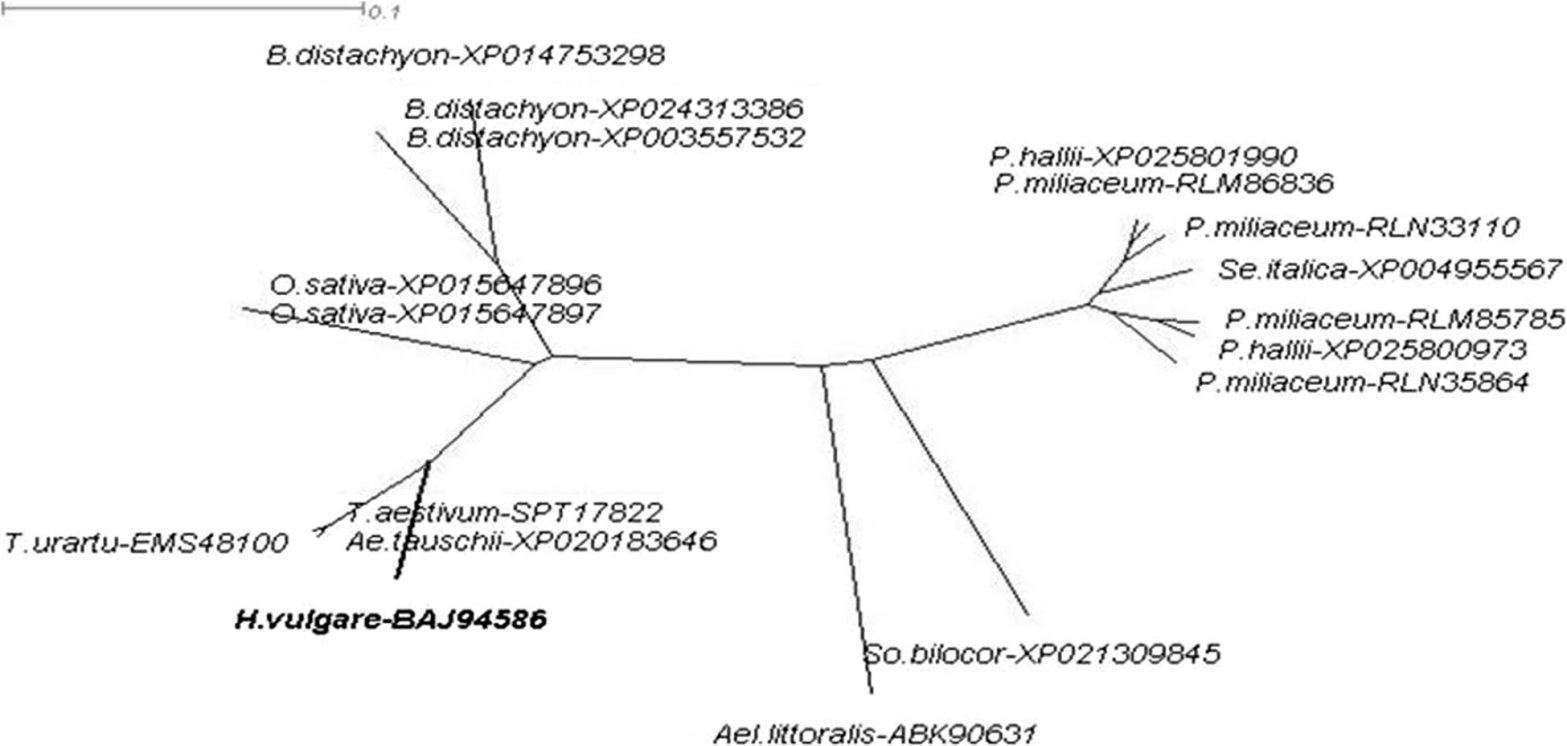
Molecular phylogenetic tree with the identified HvSAP12 protein (indicated in Bold). Homologous protein sequences from other monocot plant species were available in the NCBI database. Unrooted Consensus tree with Equal angle dendrogram was generated by the program SplitsTree4 (http://www.splitstree.org). The botanical names of the presented species are as follows, in clockwise order: *Hordeum vulgare*, *Triticum urartu*, *Aegilops tauschii*, *Triticum aestivum*, *Oryza sativa*, *Brachypodium distachyon*, *Panicum halli*, *Panicum miliaceum*, *Setaria italica*, *Sorghum bicolor* and *Aeluropus littoralis*. The corresponding protein sequence accession IDs were added after the species names

To better define the similarity between the encoded protein HvSAP12 (accession BAJ94586) with SAP protein sequences from other monocot plant species, a phylogenetic dendrogram was produced based on available monocot protein sequences in the NCBI database (https://www.ncbi.nlm.nih.gov) (Fig. 2).

### Analysis of *HvSAP* gene expression using semi-quantitative RT-PCR

**Fig. 3:**
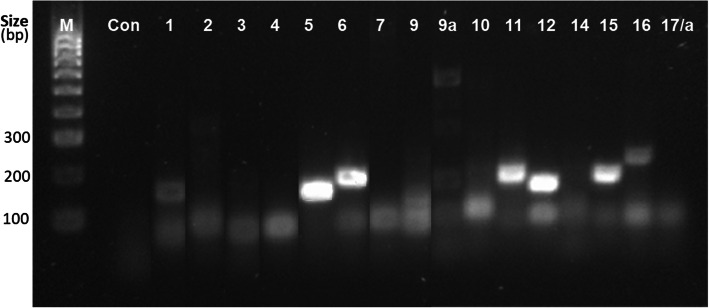
Expression of 17 *HvSAP* genes (shown at the top) detected by semi-quantitative RT-PCR. cDNA was synthesised from leaves of plants of the four cultivars grown under salt stress and compared to parental Controls (Con). M, 100 bp DNA ladder (Bioline)

Semi-quantitative RT-PCR analysis of 17 identified *HvSAP* genes with bulked cDNA samples from stressed plants revealed five highly expressed genes: *HvSAP5*, *HvSAP6*, *HvSAP11*, *HvSAP12* and *HvSAP15* (Fig. 3). Two genes, *HvSAP1* and *HvSAP16*, showed less intense but still clear expression levels, while the remaining *HvSAP* genes showed no or very poor expression, including multiple bands for the *HvSAP9a* gene. The five highly expressed genes were selected for further study.

### Expression analysis of five selected *HvSAP* genes

**Fig. 4:**
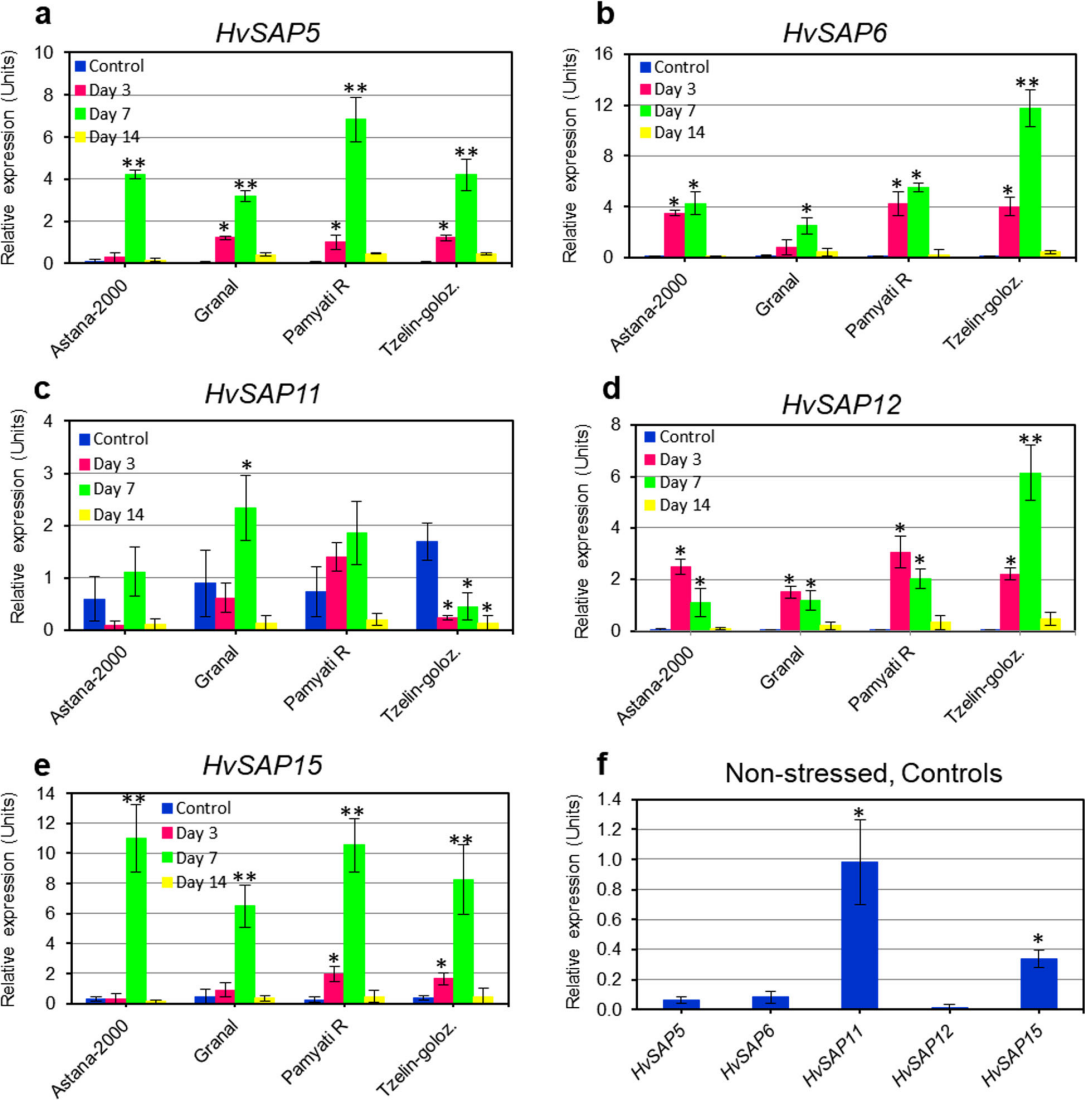
Expression analysis of five selected *HvSAP* genes. **a** *HvSAP5*; **b** *HvSAP6*; **c** *HvSAP11*; **d** *HvSAP12*; **e** *HvSAP15*, in leaves of four barley cultivars grown in hydroponics with 150 mM NaCl solution for 0 (Control), 3, 7 and 14 days. **f** Averaged expression of the five selected genes in Control plants of four studied barley accessions for comparison. Expression data were normalised using the averages of two reference genes, *HvADP* and *HvGAPDH*, and presented as the average ± SE of three biological and two technical replicates for each genotype and treatment. Significant differences are indicated by asterisks compared to Controls within each experiment, and for each barley accession (**a-e**) and among studied genes (**f**): * *P* > 0.95; ** *P* > 0.99, calculated using ANOVA

Four out of five *HvSAP* genes examined had relatively high or very high expression after 3 days and especially after 7 days exposure to salt stress (Fig. 4). The greatest increase in transcript levels were found for *HvSAP5* (7-fold), *HvSAP6* (12-fold), *HvSAP12* (6-fold), and *HvSAP15* (11-fold)*.* Two genes, *HvSAP6* and *HvSAP12* (Fig. 4b and d), showed similar expression levels on Days 3 and 7 in three out of four of the studied cultivars, while transcripts of *HvSAP5* and *HvSAP15* (Fig. 4a and e) were significantly higher on Day 7 in all four studied barley genotypes. In Control (non-stressed) plants, the expression level of the four genes varied from very low (0.35 Relative expression units in *HvSAP15*) to extremely low in *HvSAP5*, *HvSAP6* and *HvSAP12* (Fig. 4f). Only a single gene, *HvSAP11* (Fig. 4c), had an expression profile very different from the other four studied *HvSAP* genes. Firstly, the initial level of *HvSAP11* expression varied between 0.5 and 1.7 Relative expression units in all four studied cultivars (Fig. 4c), which was significantly higher than recorded in the other four *HvSAP* genes (Fig. 4f). Under salt stress, expression of *HvSAP11* was significantly (2.5-fold) increased at Day 7 in Granal, but was down-regulated during all periods of the experiment (3–14 days) in Tzelinniy golozerniy (Fig. 4c). It is too premature to draw any conclusions about correlations between the productivity of the four studied barley cultivars in salt- and drought-affected environments and the presented expression profiles of the five studied *HvSAP* genes.

### Genotyping of the segregating population Granal × Baisheshek for the *HvSAP12* gene using an Amplifluor SNP marker

**Fig. 5:**
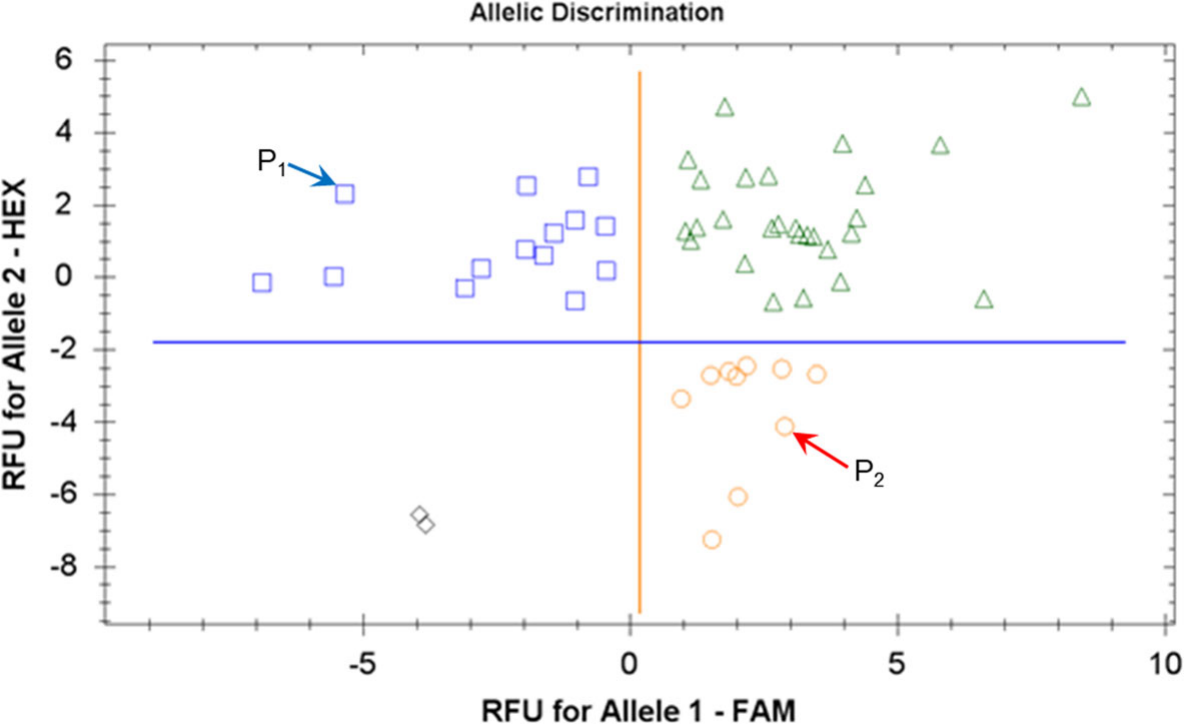
Allele discrimination for the Amplifluor-like SNP marker KATU-B30. Fifty F_3_ breeding lines from the segregating population Granal × Baisheshek were used. Parental genotypes are designated as: ♀ P_1_, Granal, and ♂ P_2_, Baisheshek. Relative Fluorescence Units (RFU) for fluorophores FAM and HEX were transformed automatically into genotyping of alleles 1 and 2, respectively, using a BioRad CFX96 Real-Time PCR Detection System Instrument

A single clear SNP was identified in the 3′-UTR region of the *HvSAP12* gene in Granal and Baisheshek, parents of the F_3_ segregating population (Additional file 1, Fig. S1). Based on this SNP, the Amplifluor-like SNP marker KATU-B30 was developed and used to score 50 breeding lines produced from the F_3_ progeny of this cross. The genotyping results using the KATU-B30 SNP marker showed good discrimination of alleles identical to those in the parents (Fig. 5). The genotyping results of *HvSAP12* were used for further comparison with plant phenotypes.

### Phenotyping and grain yield performance of F_3_ breeding lines from the segregating population Granal × Baisheshek

**Fig. 6:**
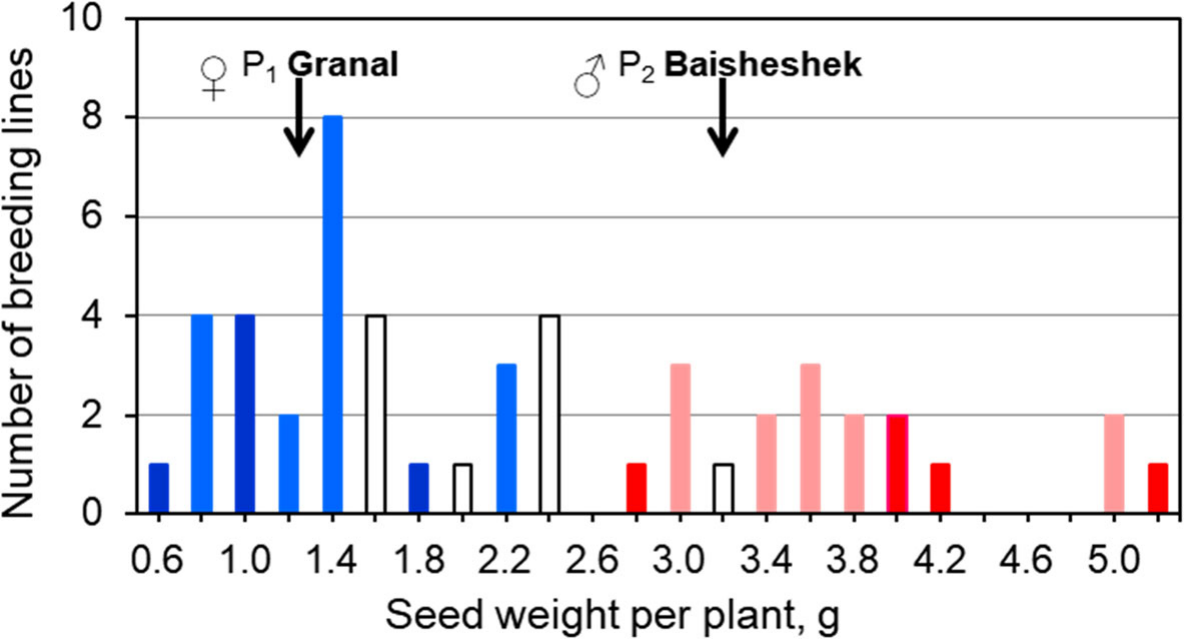
Histogram of seed yield from 50 F_3_ breeding lines produced from the segregating population Granal × Baisheshek. The breeding lines were tested in field trials in Northern and Central Kazakhstan during the dry seasons of 2017–2018 with a low-moderate level of soil salinity. Yield of the parents, ♀ P_1_, Granal, and ♂ P_2_, Baisheshek, is indicated at the top by arrows. Dark blue and dark red bars indicate completely homozygous breeding lines for the KATU-B30 SNP marker, while light blue and pink bars represent mixed breeding lines containing homo- and heterozygotes alleles 1 and 2 of the SNP marker, respectively. Clear bars relate to heterozygotes of KATU-B30 SNP

In field trials affected by drought and a low-moderate salinity level over two years, phenotyping analysis of grain yield performance of 50 breeding lines produced from F_3_ progeny showed a continuous distribution (Fig. 6). However, groups of genotypes scoring differently for the KATU-B30 SNP marker were clearly distinguished between the lowest and highest grain yields (< 2.6 and > 2.6 g per plant, respectively), and did not overlap (Fig. 6). The two groups of breeding lines with lowest and highest grain yields included homozygotes (indicated in dark blue and in red, respectively, in Fig. 6), and a mixture with heterozygotes (shown in light blue and pink).

Alleles of *HvSAP12*, derived from maternal parent Granal and designated as blue squares in Fig. 5, were associated with lower yielding breeding lines as indicated by dark and light blue bars in Fig. 6. Respectively, *HvSAP12* alleles originating from the paternal parent Baisheshek are indicated with red circles (Fig. 5) and by red and pink bars in genotypes with higher grain yields per plant (Fig. 6). A very strong association (R^2^ = 0.85) was found between *HvSAP12* genotyping (Fig. 5) and grain yield phenotyping (Fig. 6).

## Discussion

A large family of *HvSAP* genes was identified for the first time and their expressions were characterised in barley plants, in both hydroponic experiments with control (non-stressed) and NaCl treatment, as well as in field trials in a dry environment with low-moderate salinity.

There is very little published information concerning *SAP* gene expression in different plant species grown under favourable conditions. In tomato, *SlSAP* genes show much stronger similarity to *Arabidopsis* than monocot plant species; nonetheless, a comparison of tomato with barley *HvSAP* transcripts is noteworthy. Very high expressions of *SlSAP1* and *SlSAP10* were found in 9-day-old tomato seedling without any stress [6]. These genes are closely related to *OsSAP1*, *OsSAP11* and *OsSAP15* in rice, which perfectly matches our results for *HvSAP11* and *HvSAP15*, which showed the highest transcript levels in non-stressed barley plants (Fig. 4f). Two other barley genes identified in our study, *HvSAP5* and *HvSAP6*, showed smaller but still recognisable levels of gene expression in plants grown in favourable conditions. Therefore, our results are similar to those in tomato, where *SlSAP4*, *SlSAP5* and *SlSAP9* were clustered in the same clade of the phylogenetic tree [6].

In our study, five out of 17 identified *HvSAP* genes showed high expression profiles under salt stress, while other *HvSAP* genes had no or very low transcript levels. For comparison, in rice, all five orthologue genes were up-regulated in response to salinity stress [5]. The highest level of 3.5-fold increased expression was reported for *OsSAP5* and *OsSAP12*, followed by *OsSAP6* and *OsSAP11* (3-fold), and *OsSAP15* (1.5-fold). In general, our results for the five *HvSAP* genes show a similarity to those published for rice, except that *HvSAP15* compared to *OsSAP15* transcripts showed very high (6–11-fold) and relatively small (1.5-fold) expression in barley and rice, respectively.

Nevertheless, more conflicting observations were made in the comparison between other *OsSAP* and *HvSAP* genes. Three genes, *OsSAP7*, *OsSAP10* and *OsSAP14*, showed 5.5-, 9- and 10-fold up-regulation in rice seedling under salt stress [5], while orthologous barley genes (*HvSAP7*, *HvSAP10* and *HvSAP14*) in our study did not show any amplification with bulked cDNA in semi-quantitative RT-PCR, and as a result were excluded from further study. There are several possible reasons for such notable differences in orthologous gene expression. Firstly, four barley cultivars were used in our study, which is significantly more representative compared to the single rice accession published by Vij and Tyagi [5]. In that experiment, rice seedlings were grown to 7 days in trays lined with wet cotton prior to NaCl application, while barley plants in our experiment were three weeks old and grown in hydroponics with Growth solution. The rice seedlings were exposed to 200 mM NaCl for a very short time (6 h), while leaves from barley plants in our experiments were sampled after 3, 7 and 14 days, as a long-term salinity treatment with 150 mM NaCl.

However, from our point of view, the major important difference between the experiments with rice [5] and our experiments with barley is methodological, which can dramatically change the interpretation of the results. Although it was not explicitly written, we can presume that rice seedlings were simply transferred from the wet tray into a container with 200 mM NaCl and exposed for 6 h [5]. Such a sudden transfer (in one step) of plants from non-stressed conditions into solution with 200 mM NaCl would likely cause ‘osmotic shock’ and cell plasmolysis, with protoplasts detaching from the cell wall, particularly in roots. Therefore, the gene expressions reported (including *SAP* genes) were possibly the result of ‘salt shock’ rather than ‘salt stress’ as occurs with gradual (in several steps) NaCl application, as described earlier [25]. In our experiments with barley plants, high transcript levels of five genes (*HvSAP5*, *HvSAP6*, *HvSAP11*, *HvSAP12* and *HvSAP15*) were found after gradual elevation of NaCl concentration in Growth solution, and these genes indeed were responsive to salt stress. In contrast, the three reported genes in rice, *OsSAP7*, *OsSAP10* and *OsSAP14* [5], were possibly responding to the strong osmotic/salt shock rather than salt stress and therefore these results should be interpreted with regards to the method of NaCl application.

A similar situation arises for the comparison of *HvSAP* gene expression in barley with *SlSAP* genes in 9-day-old tomato seedlings transferred into media with 200 mM NaCl for 1 and 8 h [6]. All five *HvSAP* genes identified as responsive to gradual salt stress have similarity with the corresponding genes in tomato, while the group including *SlSAP6*, *SlSAP12* and *SlSAP13* genes were likely expressed in response to salt shock.

In general, absolute expression levels are not always a reliable indicator of the importance of gene function. However, the studied *HvSAP* genes showed higher or lower expression profiles (more or less transcript) in response to salt stress. Further investigation can prove which of these candidate genes are functionally more important for plant responses to stress. Nevertheless, it seems unlikely that studied genes recording only low expression and a small amount of transcript in response to salinity could have a more important functional role in plant tolerance.

Salinity and dehydration tolerance have a common response component - osmotic regulation in cells. Therefore, it is not surprising that most *SAP* genes show similar trends in co-expression in response to either salinity or rapid dehydration in detached (desiccated) rice and tomato leaves [5, 6]. In the natural environment, drought and salinity also often accompany each other. In this regard, one of the more highly expressed genes in barley, *HvSAP12*, seems to be involved in the synchronised reaction of plants to drought and salinity, particularly in field trials. In the literature, there is only one study, in wheat, which reports a strong association of a haplotype with beneficial alleles of *TaSAP1* with agronomically important traits such as grain weight, components of spike productivity and grain yield per plants under drought and in well-watered conditions [9]. Therefore, our results on the association, using 50 barley breeding lines from F_3_ progenies of a G × B cross, between the KATU-B30 SNP marker for *HvSAP12* and their grain yield per plant phenotype in dry and salt-affected field trials are similar and now add to the findings presented for bread wheat by Chang et al. [9].

However, the clusters used for allele discrimination for the KATU-B30 SNP marker were quite loosely grouped in our study, which is typical for self-developed Amplifluor-like SNP markers. Nevertheless, the alleles automatically determined by the qPCR instrument software were verified manually and compared to genotypes of the parents to ensure confidence in the results.

Marker-assisted selection based on well-established and experimentally-verified functional molecular markers can be effective for selecting the best genotypes in bread wheat plants grown in drought-prone environments [9] and in barley plants under drought and salinity stress (current study). Therefore, application of molecular markers like the KATU-B30 SNP developed here, can help to speed-up the breeding process in barley for improvement of grain production in the harsh stress-affected environment of Kazakhstan, and potentially many other countries.

## Conclusions

In barley, 17 *HvSAP* genes encoding Stress-associated proteins were identified in our study. Five genes, *HvSAP5*, *HvSAP6*, *HvSAP11*, *HvSAP12* and *HvSAP15*, were found to be highly expressed in leaves of barley plants in response to salt stress in hydroponics compared to controls, using both semi-quantitative RT-PCR and qPCR analyses. SNP marker KATU-B30 was developed for *HvSAP12*. This and other *HvSAP* genes play a definite role in the tolerance of barley plants to salinity and drought, with an association with higher grain yield shown in field trials. Marker-assisted selection with SNP marker KATU-B30 can be applied in barley breeding to improve grain yield production under conditions of abiotic stress.

## Material and methods

### Plant material

Four barley cultivars from Kazakhstan developed for animal feed production were used in this study: Astana-2000, a standard barley cultivar in Kazakhstan with high grain yield and tolerance to drought and salinity; Granal, a parent of the segregating population with elite grain quality [26] and relatively sensitive to abiotic stresses; Pamyati Raisi, an elite high-yielding tolerant barley feed cultivar in Kazakhstan; and Tzelinniy golozerniy, a hull-less or ‘naked-seed’ local cultivar producing fewer grains under drought and salinity. Based on previous results, an F_2_ hybrid population was produced from the cross between barley cultivars Granal and Baisheshes (high-yielded in dry conditions), with manual emasculation, isolation and controlled pollination conducted, and provided by Grigory Sereda (A.F. Khristenko Karaganda Agricultural Experimental Station, Karaganda Region, Kazakhstan). Seeds of barley cultivars and the propagated F_2_ segregating population were provided for this study by G. Sereda in the framework of the International collaborative research project. A small number of seeds can be provided to researchers upon request.

### Salinity stress in hydroponics and collection of leaf samples

Seeds were germinated on wet filter paper in Petri-dishes at room temperature for five days and seedlings were transferred into a hydroponics set-up using the described method [27] with the following modifications. Four tubs, each with a 12 L capacity, were covered with lids drilled with holes 1 cm in diameter. A foam piece was gently wrapped around the middle of the transferred seedling for support, enabling plants to be secured in the holes with their roots suspended in the Growth solution. Constant aeration of the media was provided by aquarium pumps. Further details, including the composition of the Growth solution and an image of the hydroponics set-up with growing plants are presented in (Fig. 5a and b in [28]). The pH of the hydroponic solution remained in the near neutral range (pH = 6.5–7.0) throughout the experiment, as confirmed by regular monitoring with a pH-meter (Activon, Model 20, Adelab Scientific, Australia) with no further adjustment required. The Growth solution was topped up with water daily and replaced completely every 10 days. When plants reached three-weeks old, NaCl was added to two boxes (designated as ‘salt-stressed’), by adding 25 mM NaCl increments, twice daily, for three days, to reach a final concentration of 150 mM NaCl, to avoid sudden salt shock [25]. Supplementary CaCl_2_ was added to maintain constant calcium activity as in the initial Growth solution (0.98 mM Ca^2+^) [29]. Two tubs with Control plants were grown identically without addition of NaCl and CaCl_2_.

All leaves were sampled from three individual plants separately into 10 ml plastic tubes, making three independent biological replicates for each genotype, treatment and collection time-points at Day 0, 3, 7 and 14 after salt application. Tubes with leaf samples were immediately frozen in liquid nitrogen and kept at − 80 °C until RNA extraction.

### Field trial tests

Field trials were conducted in the research fields of S. Seifullin Kazakh AgroTechnical University, Nur-Sultan, in Northern Kazakhstan, and of A.F.Khristenko Karaganda Agricultural Experimental Station, Karaganda Region, in Central Kazakhstan, during the 2017 and 2018 seasons, with dry conditions. The salinity level was very low after seed sowing into wet soil (EC = 1–3 dS/m) but gradually increased during the dry growing season until it reached a mild level (EC = 4–6 dS/m). Total rainfall was 107–130 mm during the vegetative growth period, lower than the average of 150–166 mm that was observed over many previous years in these regions. Two-row plots were sown, 1 m in length with 5 cm between plants in rows and 20 cm between rows. Four randomised replicates were used in each field trial over the two years of testing. Total grain yield was measured for all plants harvested in each plot and re-calculated as grams per plant.

### Identification of the ‘Gene of Interest’ using bioinformatics and molecular phylogenetic comparative analysis

The barley SNP database (http://bioinf.scri.ac.uk/barley_snpdb) was used to search and select a single target gene or ‘Gene of Interest’ for further research. BLAST analysis of the genetic fragments containing the SNP was applied to identify the full-length target gene using the Nucleotide collection of barley in the NCBI database (https://blast.ncbi.nlm.nih.gov).

Bioinformatics methods were applied to identify the full-length nucleotide sequence of *HvSAP*, and its corresponding polypeptide sequence was used for both BLASTN and BLASP in NCBI and in the IPK Barley BLAST Server (https://webblast.ipk-gatersleben.de/barley_ibsc/viroblast.php). A comparison with the rice genome was made using the Rice Gene Annotation web-site (http://rice.plantbiology.msu.edu/annotation.shtml). Chromosome locations of all *HvSAP* genes in the barley genome were found using PGSB, Plant Genome and Systems Biology, Barley Project web-site (http://pgsb.helmholtz-muenchen.de/plant/barley/fpc/searchjsp/index.jsp) and checked with the IPK Barley BLAST Server.

The molecular dendrograms of both nucleotide sequences in *SAP* genes and amino acid sequences in SAP polypeptides from barley and other monocot plants were constructed using the SplitsTree4 program (http://www.splitstree.org) [30]. The algorithm of Unrooted Consensus tree and Equal angle dendrogram option was used for preparation of both *SAP* genes and SAP polypeptide phylogenetic trees.

### RNA extraction, cDNA synthesis, semi-quantitative PCR and qPCR analysis

Three individual plants of each cultivar were selected from each hydroponic tub (salt stress and Controls) for each collection time-point. Frozen leaf samples were ground in 10-ml tubes with two 9-mm stainless steel ball bearings using a Vortex mixer. TRIzol-like reagent was used for RNA extraction, following the protocol described earlier [27] with a subsequent RNA quality check through electrophoresis of 1 μl of each RNA sample on a 1.5% agarose gel and quantification of RNA on a NanoDrop (ThermoFisher, USA). All cDNAs were synthesised from 2 μg of each RNA sample that passed quality control, after 1 μl of DNase treatment (NEB Biolab, England) with a 15 min incubation at room temperature (22 °C), and the use of Protoscript-II Reverse Transcriptase kit (NEB Biolab, England) following the manufacturer’s instructions. The quality of all cDNA samples was confirmed by PCR with products generated of the expected size.

Samples of cDNA diluted with water (1:5) were used for both semi-quantitative and qPCR analyses. For semi-quantitative RT-PCR, 1 μl of cDNA was collected from each synthesized samples and from four cultivars, including salt treatments and Controls and bulked together into one tube. Regular PCR was performed in a 15 μl reaction containing 1 μl of bulked cDNA, 1 × supplied Reaction buffer, 1.8 mM MgCl_2_, 0.2 mM each of dNTP, 0.25 μM of each primer and 1.0 unit of Go-Taq DNA polymerase (Promega, USA). Amplification was carried out in a Thermal MyCycler (BioRad, USA) with the following program: 94 °C for 2 min; 30 cycles of 94 °C for 10 s, 55 °C for 10 s, and 72 °C for 15 s; and with a final extension at 72 °C for 1 min. The amplicon size of PCR products varied between 136 and 165 bp (Additional file 1, Table S1). PCR products were separated by electrophoresis in 1.5% agarose gels stained with GelRed (Biotium, USA) with a 100 bp DNA Ladder (Bioline, USA), and visualized under UV light using a GelDoc system (BioRad, USA).

For qPCR expression analysis, a Real-Time qPCR system, Model CFX96 (BioRad, USA) was used following the qPCR protocol described earlier [31]. The total volume (10 μl) of qPCR in each well included 5 μl of 2 × KAPA SYBR FAST (KAPA Biosystems, USA), 4 μl of diluted cDNA, and 1 μl of two gene-specific primers (3 μM of each primer; Additional file 1, Table S1) as per the manufacturer’s recommendation. Expression data for the target genes were calculated with normalisation of gene expression relative to the average expression of the two reference genes: ADP-ribosylation factor 1-like protein (*HvADP*), AJ508228, and Glycolytic glyceraldehyde-3-phosphate dehydrogenase (*HvGAPDH*), X60343 [32]. At least three biological and two technical replicates were used in each qPCR experiment.

### DNA extraction and SNP Amplifluor genotyping

Five uniform plants were selected from each cultivar and breeding line from the segregating population grown in field trials, and the youngest fully-developed leaves were collected into 10-ml plastic tubes separately (non-bulked), representing five biological replicates for each cultivar and breeding line. DNA was extracted from leaf samples with phenol-chloroform as described in our earlier papers [33]. One μl of total genomic DNA was checked on a 0.8% agarose gel to assess quality, and concentration was measured by Nano-Drop (ThermoFisher, USA).

Amplifluor-like SNP analysis was carried out using a CFX96 Real-Time PCR Detection System (BioRad, USA) using DNA samples as described previously [21, 34] with the following adjustment for barley genotyping. Each reaction with a total volume of 10 μl contained: 3 μl of template DNA adjusted to 20 ng/μl, 1 μl of the two fluorescently-labelled Universal probes mix (0.125 μM each), 1 μl of allele-specific primer mix (0.075 μM of each of two forward primers and 0.39 μM of the common reverse primer), and 2 μl of 5 × Go-Taq Master-mix (Promega, USA) with the following final concentration of components: 1.75 mM MgCl_2_, 0.2 mM of dNTP and 0.05 units of Go-Taq polymerase (Promega, USA). The annotated SNP sites were used to design allele-specific primers. Sequences of the Universal probes and primers, and sizes of amplicons generated are presented in Additional file 1 (Fig. S2).

PCR was conducted using a program adjusted from those published earlier [21, 35]: initial denaturation, 95 °C, 2 min; 20 ‘doubled’ cycles of 95 °C for 10 s, 60 °C for 10 s, 72 °C for 20 s, 95 °C for 10 s, 55 °C for 20 s and 72 °C for 50 s; with recording of allele-specific fluorescence after each cycle. Genotyping by SNP calling was determined automatically by the instrument software, but each SNP result was also checked manually using amplification curves and final allele discrimination. The experiments were repeated twice with two technical replicates, confirming the confidence of SNP calls.

### Statistical analysis

IBM SPSS Statistical software was used to calculate and analyse means and standard error using ANOVA, and to estimate the probabilities for significance using Student’s *t*-test. A correlation analysis R^2^ was performed using Tests of Between-Subjects Effects (IBM SPSS, Statistics Desktop 25.0.0.0).

## Supplementary information

**Additional file 1: Figure S1.** Fragments of sequences in 3′-UTR region of *HvSAP12* gene in two barley parents, Granal and Baisheshek. **Figure S2.** Position of SNP, primer design, sequences of the Universal probes and SNP-specific primers, and size of amplicons. **Table S1.** Primers used for semi-quantitative RT-PCR and qPCR analysis of 17 *HvSAP* genes and two Reference genes including amplicon sizes and oligonucleotide sequences.

## Data Availability

All generated and analysed data during this study are included in this published article and its additional files.

## Abbreviations

KATU: Kazakh AgroTechnical University
SAP: stress associated protein
SNP: single nucleotide polymorphisms
TNF: tumor necrosis factor
