# Supplementary material for: Identification, gene expression and genetic polymorphism of zinc finger A20/AN1 stress-associated genes, HvSAP, in salt stressed barley from Kazakhstan
Source: BMC Plant Biol. 2020 Oct 14;20(Suppl 1):156. doi: 10.1186/s12870-020-02332-4 (PMC7556924; doi:10.1186/s12870-020-02332-4)
Supplement: Supplementary file 1 — Additional file 1: Figure S1. Fragments of sequences in 3′-UTR region of HvSAP12 gene in two barley parents, Granal and Baisheshek. Figure S2. Position of SNP, primer design, sequences of the Universal probes and SNP-specific primers, and size of amplicons. Table S1. Primers used for semi-quantitative RT-PCR and qPCR analysis of 17 HvSAP genes and two Reference genes including amplicon sizes and oligonucleotide sequences. [file 12870_2020_2332_MOESM1_ESM.pdf]

Additional file 1

Baidyussen A. et al. Identification, gene expression and genetic polymorphism of Zinc finger A20/AN1 stress-associated genes, *HvSAP*, in salt stressed barley from Kazakhstan

Figure S1. Fragments of sequences in 3'-UTR region of *HvSAP12* gene in two barley parents, Granal and Baisheshek. Positions of the identified SNP is indicated on the top by arrows.

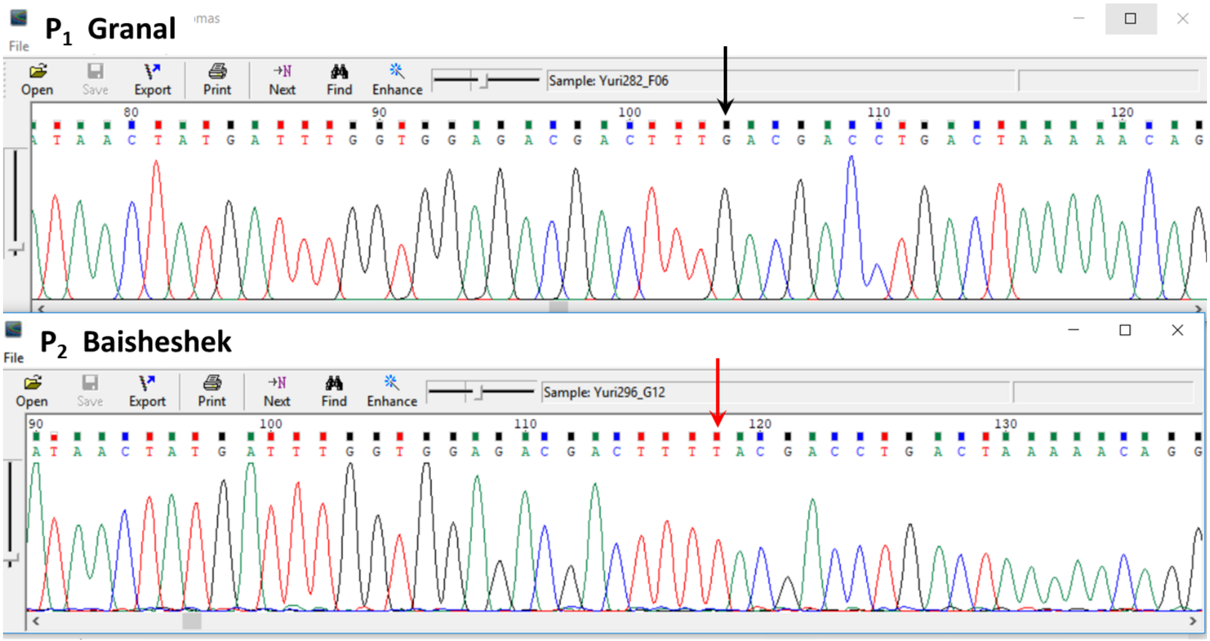

Figure S2. Position of SNP, primer design, sequences of the Universal probes and SNP-specific primers, and size of amplicons.

CTCATTCAGCAAACACTAGCCGTGATAGAGGAAGAGTCCCGATATTTCAATGAGATGATAAC
TATGATTTGGTGGAGACGACTTTKACGACCTGACTAAAAACAGGAATGATGTGAAGCCTTAG
CAATCGTCAAACCAACTCCAAGTGGTTATTGACCACGCCGGAGTACGATCAACCTGACCACG
AAGGTCTCTTCCTGCAAGCAGTCAAAGAACAAACAATAGTATGATAAAGCAATCTGAATATT

KATU-B30-SNP-F7: GAAGGTGACCAAGTTCATGCT**GATTTGGTGGAGACGACTTTG**
(Granal allele)

KATU-B30-SNP-F8: GAAGGTCGGAGTCAACGGATT**ATGATTTGGTGGAGACGACTTTT**
(Baisheshek allele)

KATU-B30-SNP-R2: GTCAATAACCACTTGGAGTTGG

Amplicon size = 93 bp

Sequence of two Universal probes

Universal probe 1:
5′-FAM-AGCGATGCGTTCGAGCATCGC(T*-BHQ1)*GAAGGTGACCAAGTTCATGCT*-3′

Universal probe 2:
5′-HEX-AGGACGCTGAGATGCGTCC(T*-BHQ1)*GAAGGTCGGAGTCAACGGATT*-3′

Table S1. Primers used for semi-quantitative RT-PCR and qPCR analysis of 17 *HvSAP* genes and two Reference genes including amplicon sizes and oligonucleotide sequences. The last two genes *HvSAP17* and *HvSAP17a* have common primers due to insufficient differences in the gene sequences. Five genes highlighted in yellow showed high expression in the semi-quantitative RT-PCR and were selected for further qPCR. The information about two Reference genes was taken from Ferdous et al., 2015 [32].

Entry	Name of gene/primer	Amplicon (bp)	F / R	Sequence (5' - 3')
1	HvSAP1-qPCR	146	F	GCGAAGCCGGCGAGGACG
			R	GCCTTGTAGTCGTAGCTGCACC
2	HvSAP2-qPCR	156	F	CAAGTGCTTCCGCGACAGCCT
			R	CCCATCAGGCCCACCTTCC
3	HvSAP3-qPCR	160	F	GACACCCGAGAAGACGAAGGC
			R	GCCTTGTAGTCGAAGAAGCAGGT
4	HvSAP4-qPCR	235	F	AAGGAGATGGTCATGAAGGAGGAA
			R	CGATTCGGCCCCTCCTTCCT
5	HvSAP5-qPCR	136	F	CCTCCTCTTTTGACAGCATCGTC
			R	GGACCAGCGATGTCAGCAGG
6	HvSAP6-qPCR	165	F	GTGGCAGAGATGAAGGATGAAGC
			R	GTGCATCGAGCAGAAGGTGTCT
7	HvSAP7-qPCR	206	F	CAGCTGACCCCGCCGTGA
			R	GACGGGGTTGTGCTTGGCTATT
8	HvSAP9-qPCR	219	F	GCTGCAGCTGGTGCTGATGCT
			R	AAGAGGGTTCTGCTGCGCCATCA
9	HvSAP9a-qPCR	205	F	TGCGGCGGCCTGCGTCGA
			R	CAGAGTCCTGCAGGCTCAGC
10	HvSAP10-qPCR	156	F	CACCACCGGCAACATGTGCTCT
			R	CACGCACCGGCTCGCCCT
11	HvSAP11-qPCR	163	F	CGTTCCCGCTCTTCGACAAGC
			R	AACCCCGTCAGGCCCACG
12	HvSAP12-qPCR	139	F	CAAAGCCGCCCAGCAACCGA
			R	GCCTTCTTGTAGTCGAATGAGCAT
13	HvSAP14-qPCR	137	F	TGGGGCTGCTGGGGTTCCT
			R	GATCACGATGGGGTTGTGCTTGA
14	HvSAP15-qPCR	160	F	CATTGTCGTCTGTGCTGTTCGTT
			R	TCCTCCCGACCCACAGTTTTATAA
15	HvSAP16-qPCR	208	F	AGTTCCTGGGTGCAGAGAAGCA
			R	GGAAGAACTACTGCTACTGCTGTT
16	HvSAP17/17a-qPCR	130	F	ACAAGACGGACGCGGAGATC
			R	TGGCTGGTGTTGGAGAAGGTGA
17	HvADP	77	F	GCTCTCCAACAACATTGCCAAC
			R	GAGACATCCAGCATCATTCATTCC
18	HvGAPDH	78	F	GCCAAGACCCAGTAGAGC
			R	CACATTTATTCCCATAGACAAAGG
